# Advances in Research on the Mechanism of Heterosis in Plants

**DOI:** 10.3389/fpls.2021.745726

**Published:** 2021-09-27

**Authors:** Xilin Wu, Yan Liu, Yaowei Zhang, Ran Gu

**Affiliations:** ^1^Key Laboratory of Biology and Genetic Improvement of Horticultural Crops (Northeast Region), Ministry of Agriculture and Rural Affairs, Harbin, China; ^2^College of Horticulture and Landscape Architecture, Northeast Agricultural University, Harbin, China

**Keywords:** heterosis, plant, biomass yield, grain yield, gene

## Abstract

Heterosis is a common biological phenomenon in nature. It substantially contributes to the biomass yield and grain yield of plants. Moreover, this phenomenon results in high economic returns in agricultural production. However, the utilization of heterosis far exceeds the level of theoretical research on this phenomenon. In this review, the recent progress in research on heterosis in plants was reviewed from the aspects of classical genetics, parental genetic distance, quantitative trait loci, transcriptomes, proteomes, epigenetics (DNA methylation, histone modification, and small RNA), and hormone regulation. A regulatory network of various heterosis-related genes under the action of different regulatory factors was summarized. This review lays a foundation for the in-depth study of the molecular and physiological aspects of this phenomenon to promote its effects on increasing the yield of agricultural production.

## Introduction

Heterosis is a common biological phenomenon in nature. It refers to the heterozygote produced by hybridization between two or more parents with different genetic bases. Hybrids are superior to parents in terms of yield, growth rate, viability, and disease resistance (Hochholdinger and Hoecker, 2007). In agricultural production, heterosis is widely utilized in animals, such as silkworms, and plants, such as rice, maize, vegetables, and some perennials (Vaillancourt et al., 1995; Arcade et al., 1996; Kopp et al., 2002; Marcelo et al., 2007; Costa et al., 2014; Chen et al., 2020; Wang et al., 2021; Yu et al., 2021; Zhang et al., 2021). The use of heterosis has gradually improved the yield, quality, and disease resistance of animals and plants, thereby enhancing the social and economic benefits of agricultural production. However, even after over 100 years of research, the mechanism by which heterosis forms remains obscure. Therefore, although heterosis is widely utilized, our theoretical understanding of this phenomenon is incomplete. Moreover, limited research on the genetic basis of heterosis restricts its further applications in agricultural production. After Darwin (1876) was the first to propose the idea of heterosis after he noted that the yield of hybrid generations produced by inbred maize hybrids was 25% higher than that of their parents. Since then, several hypotheses have been offered to explain its genetic basis. Heterosis is a complex physiological and biochemical genetic phenomenon that scientists have been trying to understand from different aspects, and have obtained some relevant progress, such as classical genetics, molecular genetics, epigenetics, and physiology. Here, the recent researches on plant heterosis were summarized from different aspects including classical genetics, parental genetic distance (GD), quantitative trait loci (QTL) transcriptomes, proteomes, epigenetics, hormone regulation and gene regulation, and can provide theoretical reference for improving plant yield in breeding in the future.

## Plant Heterosis According to Classical Genetics

Prior to the advent of modern molecular biology and molecular genetics, the mechanism of heterosis was explained by three primary hypotheses based on classical genetics. The first is the dominant hypothesis, which emphasizes the dominant complementarity of favorable alleles in hybrid lines; this hypothesis states that the harmful genes of one parent can be covered by the favorable dominant genes of the other parent. This hypothesis was first proposed by Bruce (1910). Jones (1917) further improved this hypothesis and proposed the dominance of linked genes hypothesis. Analysis of the heterozygosity and detection of the QTL of 141 restriction fragment length polymorphisms revealed that the heterozygosity of rice heterosis is not related to the heterozygosity of its whole genome. Xiao et al. (1995) reported that the comprehensive performance of recombinant lines screened in F8 generations is better than that of inbred lines, further proving that the dominant effect of parental genes substantially affects rice heterosis. This observation supports the dominance hypothesis. Heterozygous fragments containing *qSS7* and *qHD8* exerted dominant effects that contributed to the heterosis of the hybrid rice variety “Liangyoupei9” (LYP9) (Lin T. et al., 2020). Large-scale genetic analysis of the offspring of three excellent maize hybrids and examination of the heterosis patterns of 628 related loci revealed that most loci have complete–incomplete dominant (main) or overdominant (secondary) effects on hybrid genotypes; moreover, the proportion of dominant alleles from two parental lines was almost equal, which was the main reason for the strong heterosis (Liu H. et al., 2019). The next hypotheses based on classical genetics is the superdominance hypothesis, which emphasizes that the interaction of heterozygous alleles in hybrid lines has a stronger growth heterosis than the parental homozygous alleles (Shull, 1908). A 100 years later, Krieger et al. (2010) and Guo et al. (2013) found the superdominant QTLs in tomato and cotton. Chen L. et al. (2018) analyzed the transcriptome of the development of young panicles of the rice variety WFYT025, and they argued that the superdominant effect may be the main reason for the heterosis its grain number. Differentially expressed genes at the same position as grain number QTL is considered a candidate gene that can provide valuable targets for cloning and functional analysis of these grain number QTLs. Through genome-wide comparative transcriptome analysis, Shahzad et al. (2020) found that the circadian rhythm pathway genes related to *LATE ELONGATED HYPOCOTYL* (*LHY*) and many root genes involved in peroxidase activity also show overdominant expression in hybrids. They added that the overdominance of gene expression levels plays a key role in the early biomass vigor of cotton hybrids. Tian M. et al. (2018) performed transcriptome analysis of F1 hybrids and found that the genes involved in nicotine synthesis and metabolism (*ADC*, *PMT*, *MPO*, *QPT*, *AO*, *QS*, *QPT*, *A622*, and *BBLs*) and nicotine-transport genes (*JAT2*, *MATE1*, *MATE2*, *NUP1*, and *NUP2*) are upregulated, indicating that the overdominant expression of nicotine-metabolism genes plays a key role in F1 heterosis. These results strongly proved that superdominance is an important genetic basis for heterosis. Another hypotheses based on classical genetics is the epistatic hypothesis. Powers (1944) argued that the interaction of non-alleles in hybrids at different loci is the core of heterosis; this assumption had been confirmed in the analysis of heterosis in maize and rice (Yu et al., 1997; Tang et al., 2010), especially in terms of grains per panicle and grain weight per panicle of rice (Li et al., 1997; Zhang et al., 2021). Furthermore, harmful alleles can repress other favorable QTLs by epistasis during floral transition in maize (Xiao et al., 2021). The aforementioned theories explain the genetic basis of heterosis from different angles. These hypotheses highlight the most essential genetic basis of heterosis that genetic heterogeneity is required among hybrid parents. However, the three models of dominance, superdominance, and epistasis are not mutually exclusive, and they are all related to plant heterosis. Furthermore, dividing heterosis into three independent proportions (i.e., the contribution of dominance, superdominance, or epistasis) is difficult because heterosis is a non-linear effect of multiple heterozygous gene combinations in agricultural production (Liu J. et al., 2020).

## Parental Genetic Distance and Plant Heterosis

The genetic basis of phenotypic differences between hybrids and their parents originates from differences in genomic composition. With the rapid development of the fields of molecular genetics and functional genomics, genetic differences between hybrid parents and their relationship with heterosis can now be evaluated *via* molecular analysis to determine the molecular mechanism of heterosis. Genetic differences between hybrid parents can be expressed by GD, and the degree of heterosis is strongly correlated with the GD of both parents. In a broad sense, the degree of heterosis increases with the increase of GD (Usatov et al., 2014; Boeven et al., 2020), and the farther the GD between the parent inbred lines is, the more scattered the gene expression in the hybrids will be (Ma et al., 2006; Stupar and Springer, 2006; Swanson-Wagner et al., 2006; Zhang and Borevitz, 2009; Birchler and Veitia, 2010; He et al., 2010; Paschold et al., 2012). Studies on a variety of plants have shown that GD has a considerable correlation with heterosis. In rice, Jaikishan et al. (2010) found that compared with rice genome simple sequence repetition (SSR), expressed sequence tag (EST) derived SSR (EST-SSRs) can lead to a better correlation between GD and grain yield heterosis. In addition to the rice, Sudi et al. (2010) conducted molecular, morphological, and genealogical analyses, finding that the genetic diversity of wheat is moderately to substantially correlated with plant height. Furthermore, they noted that the genetic diversity of wheat according to morphological markers is moderately to considerably correlated with grain number per spike. Hao et al. (2015) used single nucleotide polymorphism (SNP) to evaluate the genetic diversity, indicating that the subpopulation structure of waxy corn and common maize populations is related to heterosis. On the basis of SSR markers related to yield, GD, mid-parent heterosis (MPH), and best-parent heterosis (BPH) have a consistent and substantial correlation with the yields of seed cotton and lint in the Alar area (Li X. et al., 2019).

However, the experimental results of many scholars are opposite to the above views. They believed that GD estimated by molecular markers is not significant correlated with heterosis, and could not be used to predict heterosis. For example, Arcade et al. (1996) found that there is no correlation between some quantitative traits of larch and GD based on random amplified polymorphic DNA (RAPD). Rajendran et al. (2014) noted that the GD in rice is no correlated with heterosis through SSR marker. Kawamura et al. (2015) found that the GD estimated by SSR and cleaved amplified polymorphic sequence (CAPS) markers is not correlated with the heterosis of Chinese cabbage. Chen et al. (2020) found that the correlation between GD and heterosis in eucalyptus is poor by EST-SSR markers. Similarly, some studies have shown that GD also does not predict heterosis levels in maize, wheat and melon (Dreisigacker et al., 2005; Maruthi et al., 2019; Asaf et al., 2021). But Tian et al. (2017) argued that the GD estimated using random SSR and sequence related amplified polymorphism (SRAP) primers is not correlated with the heterosis of *Brassica napus*, whereas the GD estimated by the F1 heterozygous marker is highly correlated with the number of pods, thousand-seed weight, and single-species yield of this plant.

In addition, it has been reported that GD is highly positive correlation with geographical distance in *Linum austriacum* (Sheidai et al., 2014), *Trachypogon plumosus* (Baruch et al., 2004), *Sinocalycanthus chinensis* (Li et al., 2012), and *Erodium ciconium* (Xi et al., 2021). And in rough fescue, GD estimated by RAPD markers is related to its geographical distance. Genetic diversity seems to increase from west to east in Alberta, and the populations farther east have greater adaptive adaptability (Zhao et al., 2008). In addition, it has highly positive correlation between GD and geographical distance when using SRAP markers to determine the heterosis of pepper. Further studies showed that when the GD is less than 0.4051, the yield heterosis increases with the increase of GD, and when the GD is greater than 0.4051, the yield heterosis decreases with the increase of GD (Wu et al., 2012). However, Upadhyay et al. (2011) found that geographical distance do not contribute to genetic divergence in dolichos beans. This suggests that predicting levels of heterosis needs to be done within a valid geographical range. Overall, when species diversity is more abundant, they are more able to extend their geographical range and adapt better to new environments.

In summary, although the calculation of GD based on molecular markers is widely used, it cannot be applied to predict heterosis strictly and accurately. However, with the development of high-throughput sequencing technology, the application of molecular markers will be more helpful to predict heterosis. In addition, it can be found that the potential of molecular markers to predict crossing performance may largely depend on plant materials, phenotype, prediction methods and planting environment. Unless the DNA markers used in the analysis are associated with the genes affecting relative traits, hybrid performance cannot be accurately predicted by GD. Restriction-fragment length polymorphism (RFLP), SSR and other random markers may not be associated with relative traits, while other molecular markers whose primers are designed according to target genes have relatively high correlation to relative traits. So the correlation between molecular markers and heterosis cannot be accurately described.

## Quantitative Trait loci-Associated Heterosis in Plant

Various QTLs have been identified for general or specific combination ability in hybrids. In the research of rice, heterosis can be affected by genes that regulate spikelets, such as *SQUAMOSA PROMOTER BINDING PROTEIN-LIKE 14* (*SPL14*) and *Gnarley1* (Ashikari et al., 2005; Jiao et al., 2010; Miura et al., 2010). And Huang et al. (2016) detected different dominant loci in different types of hybrid rice, such as *Heading date 3a* (*Hd3a*) and *TILLER ANGLE CONTRO 1* (*TAC1*) in the hybrid rice system of cytoplasmic male sterility (CMS) for female parental lines; *LAX PANICLE 1* (*LAX1*) and *Grain number*, *plant height and heading date 8* (*Ghd8*) in the two-line system of environmentally sensitive male sterility; and *DENSE AND ERECT PANICLE 1* (*DEP1*), *IDEAL PLANT ARCHITECTURE 1* (*IPA1*), and *NARROW LEAF 1* (*NAL1*) in the system of the two populations from rice indica–japonica crosses. Li D. et al. (2016) re-sequenced the recombinant inbred lines population of the super hybrid rice LYP9. Combined with transcriptome analysis of early panicle development, they found the main heterosis locus *RH8* and photoperiod-sensitive genes *DAYS TO HEADING 8* (*DTH8*)/*Ghd8*/*LATE HEADING DATE 1* (*LHD1*). *Ghd8* is the primary gene for heterosis in most Indica–Indica hybrid systems (Huang et al., 2016) and LYP9 (Li D. et al., 2016). Another study showed that the heterosis loci on chromosomes 11 and 12 of rice have strong heterosis effects on biomass, panicle weight, grain yield, and other traits (Lin Z. et al., 2020). In addition to the study of heterosis in rice, a study that adopted different methods in different combinations and even different species showed that flowering-related genes are related to heterosis, such as the tomato flowering gene *SINGLE FLOWER TRUSS* contributes to the heterosis of tomato yield (Krieger et al., 2010). Li et al. (2011) found that the histone deacetylase genes in hybrid rice cause early flowering by regulating the expression of some non-additive genes and the key genes during flowering, such as *Ghd7* and *Hd3a*. Wang et al. (2018a) examined the heterosis of four maize core-related traits in two experimental populations of chromosome segment substitution lines derived from Reid × TSPT. They identified 63 and 57 different QTL loci from the two populations, which provided the basis for fine mapping of heterosis loci in grain size. Sarfraz et al. (2018) focused on 32 heterosis QTLs related to cotton fiber quality traits. They found 96 unique favorable alleles that are chiefly related to fiber quality. Liu C. et al. (2019) reported that triploid loquat has a more remarkable heterosis than diploid and tetraploid loquat, and they found that the heterosis of triploid loquat is related to the clock genes *TIMING OF CAB EXPRESSION 1 (TOC1)*, *LHY*, and *GIGANTEA* (*GI*). Liu Y. et al. (2020) detected six QTLs of maize kernel-related traits and the MPH of these traits. They found that the heterosis of grain size and the genetic mechanism of kernel length, kernel width, kernel thickness, and hundred-kernel weight are not completely independent. In summary, QTLs related to heterosis have a complex regulatory network, and environment, genetic background and ploidy can impact on this regulatory network. This is also one of the difficulties in heterosis research.

## Transcriptomics and Proteomics Reveal Plant Heterosis

Different genes are involved in the formation of heterosis in different tissues and developmental stages. In turn, these heterosis-related genes are involved in transcription, translation, cell division, transportation, signal transduction, defense and stress response, biological regulation, development, energy metabolism, protein metabolism, amino acid metabolism, biosynthesis of secondary metabolites, photosynthesis, carbon fixation, chlorophyll synthesis, carbohydrate metabolism, photorespiration, nitrogen absorption, and cofactor and vitamin metabolism (Bao et al., 2005; Lai et al., 2006; Meyer et al., 2007; Song et al., 2007, 2010; Zhang et al., 2008, 2012; Li et al., 2009; Fujimoto et al., 2012). Liu et al. (2021) determined that the growth heterosis of hybrids is determined by the combined ability of cell division and photosynthesis, and the early development of hybrid leaves might enhance the growth heterosis of hybrids. In the past century, some scholars proposed a multiple gene model with complementary alleles and gene expression variations, all of which may be an important factor that leads to heterosis. With the emergence of DNA microarray, QTL analysis, allele expression analysis, real-time quantitative single nucleotide site typing detection of allele fluorescence, and high-throughput sequencing technology, differences in allele expression can now be detected. Differential gene expression may be a reflection of differential gene expression in parents, differential expression of primary and secondary metabolic genes, and changes in metabolic profiles. The research of Yi et al. (2020) indicated that heterosis may related to changes in primary and secondary metabolic balance.

Preliminary studies on hybrid rice have shown that the polymorphism of differentially expressed transcription factors and promoter elements are two important factors for rice heterosis (Zhang et al., 2008). Shao et al. (2019) observed that allele-specific expression (ASE) genes were significantly enriched in the genomic regions of differential selection in the process of rice breeding, indicating that ASE is strongly affected by the expression level of the parent genes. In addition, the study on the relationship between transcriptomics and heterosis suggested that the additive and non-additive expression of genes are the main patterns that constitute the difference in gene expression between hybrids and parents. In maize hybrids, Paschold et al. (2012) found that 10% of the genes are non-additively expressed, and 14% of the genes are ASE. Li et al. (2017) examined the heterosis of traits related to panicle weight by using excellent maize inbred lines. They proved that combinations of heterosis loci depend on the genotype. To identify heterosis-related genes in *Brassica* species, Yi et al. (2017) used the Br300K microarray to perform transcriptome analysis at three developmental stages in non-heading Chinese cabbage. They observed that numerous genes were differentially expressed in F1 hybrids, with prominent non-additive expression. The genes specifically expressed in the three stages of F1 hybrids were some unidentified genes specific to *Brassica* and several genes related to defense. Zhang et al. (2016) found that the chromatin remodeler *DECREASE IN DNA METHYLATION 1* (*DDM1*) affects the heterosis of *Arabidopsis* by regulating salicylic acid metabolism, and this gene can promote the non-additive expression of related genes. However, transcriptome analysis of hybrid broccoli revealed that the entire gene expression profile of the hybrids and their parents are similar, and only a few genes showed significant differential expression levels in the hybrids and their parents (Li et al., 2018). Guo et al. (2010) found no significant correlation between maize heterosis and the frequency of non-additive expression but observed a positive correlation between maize heterosis and the proportion of additive expression. These findings were supported by the results of Meyer et al. (2012) on *Arabidopsis* Different selections among different subgroups form different heterosis alleles. The male and female parents of hybrid rice have genetic variations. When the female genome is introduced from other subspecies and shows a high level, allele differences between the male and the female parents at the heterosis loci will result in the formation of heterosis loci in hybrid rice (Lin Z. et al., 2020).

Gene expression in hybrids is affected by *cis*-acting elements, *trans*-acting factors, and their interaction (Birchler and Veitia, 2010). *Cis*-regulatory elements are short DNA sequences containing trans-factor-specific binding sites that are used to control the expression of their associated genes (Bao et al., 2019; Luo et al., 2021). It can lead to changes in gene expression, and in hybrids, different regulatory factors and transcriptional networks recombine (Birchler et al., 2003; Riddle and Birchler, 2003; Zhang et al., 2008; Bougas et al., 2010). ASE is closely related to the parental origin effect and regulated by the complex interaction of *cis*- and *trans*-acting factors, a feature considered a key factor to differences between hybrids and their parents (Botet and Keurentjes, 2020). The genome study of Zhang and Borevitz (2009) revealed that over 40% of DEGs in parents showed ASE in hybrids, indicating that *cis*-regulatory variations have an effect on hybrid loci. Liu et al. (2018b) concluded that the differences in gene expression between parental alleles in triploid loquat are largely due to *cis*-regulatory variations. Li et al. (2021) demonstrated that *trans*-regulatory factors have a greater influence on differences in parental expression than *cis*-regulatory factors. In hybrids, genes can appear as homozygous or heterozygous pairs. Homozygous genes may be differentially expressed in different genetic backgrounds, indicating that transregulated variation has an additive effect on phenotypes. By comparison, different alleles of heterozygous genes can be differentially expressed in the same genetic background, indicating that *cis*-regulation or ASE mutation occurs (Metzger et al., 2016).

At the gene expression level, many recent studies have shown that heterosis can be revealed not only at the transcriptional level but also at the proteome level. Proteomics analysis indicated that indole-3-acetic acid (IAA) content was positively correlated with the length of the eighth internode of maize, but negatively correlated with the extent of the heterosis of the length of the eighth internode (Chen Y. et al., 2018). Analysis of the proteomics of popcorn hybrid combinations revealed that 22 kinds of biological processes are related to non-additive proteins. The heterosis of the popcorn hybrid at the early stage of plant development is related to the upregulation of protein synthesis and energy metabolism (Rockenbach et al., 2018). Marcon et al. (2013) scrutinized the proteome of maize seminal roots to identify the molecular basis of development vitality of hybrid seedlings. They detected 85 proteins that non-additively accumulate in at least one hybrid, and the result indicated that the increase of hybrid protein synthesis rate might be related to the early performance of hybrid vigor in seminal roots. Wang et al. (2021) identified over 2000 proteomes from maize hybrids and their parents’ seedling leaves *via* label-free quantification. Moreover, they verified four stress-related proteins and eight photosynthetic-related proteins *via* parallel reaction monitoring. Among these proteins, 10 were substantially different from the mid-parent’s proteins. Consistent with changes in the gene expression of hybrids and allopolyploids, both additive and non-additive proteomic patterns have been found in embryos (Marcon et al., 2010), roots (Hoecker et al., 2008), and spikes (Dahal et al., 2012) of maize hybrids; mature embryos of rice hybrids (Wang et al., 2008), and leaves of *Arabidopsis* autopolyploids and allopolyploids (Ng et al., 2012). Similar non-additive proteomes have also been found in the embryos and roots of maize hybrids and embryos of rice hybrids (Hoecker et al., 2008; Marcon et al., 2010), indicating that the hybrids of different plants have common regulatory changes.

In summary, the molecular mechanism of plant heterosis is mainly related to the gene expression in the growth and development-related pathways, involving photosynthesis, transportation, nutrition, resistance and epigenetic network. A number of studies at the transcriptome level have shown that the differential expression of genes, gene additive and non-additive expression between hybrid progeny and parents are the most critical factors to explain the heterosis mechanism. In the proteome comparison between F1 and its parents, most of the differentially expressed proteins are non-additive, and the proteins are the performers and executors of gene functions. Therefore, proteomics is a necessary supplement and effective proof of transcriptomics. However, neither transcriptomics nor proteomics can fully explain the mechanism of heterosis. It is necessary to combine multi-omics research and make a comprehensive analyze from multiple levels to reveal the molecular genetic mechanism of plant heterosis.

## Epigenetics and Plant Heterosis

Given that gene expression is affected by DNA methylation, histone modification and non-coding RNA, breeders have adopted epigenetic breeding. Epigenetics refers to a phenotypic variation that is not caused by changes in gene functions due to changes in DNA sequences. Epigenetic regulation can regulate plant gene expression from three levels, namely, DNA methylation, histone modification, and non-coding RNA, all of which are closely related to the formation of heterosis. Epigenetic factors are among the key factors that determine hybrid performance. The heterosis of *Arabidopsis* hybrids can be directly or indirectly triggered by epigenetic differences between parental lines without being affected by genetic changes (Lauss et al., 2018; Rockenbach et al., 2018). Kawanabe et al. (2016) found that the heterosis of *Arabidopsis* is reduced by the knockout of *METHYLTRANSFERASE 1*, and the reduction of heterosis is highly correlated with the decrease in methylation levels of some important genes.

### DNA Methylation and Heterosis

The methylation level of DNA in hybrids is remarkably different from that of their parents, especially when the methylation level of the epiallele region of the parents is considerably different, as it often causes substantial changes in the methylation level of the hybrids. Homozygosity or heterozygosity of methylated DNA may be involved in regulating inbreeding depression or heterosis (Nakamura and Hosaka, 2010). On the basis of the sequences of different ecotypes of *Arabidopsis* hybrids and their parents, Shen et al. (2012) found that the methylation levels of hybrids generally increase, and most of them occur in regions with different parental methylation levels. They proposed that the decrease in methylation levels in hybrids may affect growth vigor. Identically, differential DNA methylation affects gene regulation and heterosis phenotypes (Liu et al., 2018a). Analysis of the heterosis of broccoli bulb yield revealed that the DNA methylation rate of hybrids is higher than that of their parents, and the loci with different methylation levels are dominant in intergenic regions (Li et al., 2018). Moreover, the degree of DNA methylation of hybrids is proportional to the degree of genetic relationship between parents. The farther the genetic relationship between parents is, the greater the change in DNA methylation degree of F1 will be (Kawanabe et al., 2016). To study epigenetic modification and its relationship with gene expression (Chodavarapu et al., 2012), sequenced the Japonica rice Nipponbare and the Indica rice 9311 and their hybrid F1. They found that 7.48% of cytosine methylation levels between Nipponbare and 9311 were different, whereas the difference between parents and F1 was only 0.79%, suggesting that the heterosis of F1 is related to its DNA methylation level. Xiong et al. (1999) found that the methylation level of rice hybrid F1 (18%) is higher than the average methylation level of its parents (16.3%). Some methylation variations change the transcription level, which play a role in the increase in biomass heterosis (Greaves et al., 2012). Wang et al. (2018b) analyzed soybean hybrids by using MSAP and found that the increase in node number is promoted by hypomethylation, and the stem diameter of hybrids can be increased by hypermethylation. More interestingly, the methylation level of alleles from both parents will change when the DNA methylation level greatly varies. For example, the hybridization of Col-0 ecotype *Arabidopsis* with low *tRNA ADENOSINE DEAMINASE 3* (*TAD3*) methylation level and Nok-1 ecotype *Arabidopsis* with high TAD3 methylation level showed that the methylation level of *TAD3* from Col-0 increased and that of *TAD3* from Nok-1 decreased in F1 (Astrid et al., 2017).

### Histone Modification and Plant Heterosis

Histone modifications include acetylation, methylation, ubiquitination, phosphorylation, glycosylation, and carbonylation. Studies on acetylation and methylation are very thorough because they are closely related to gene expression regulation. The acetylation of histone lysine is established by histone acetyltransferases and eliminated by histone deacetylases, which is usually related to gene activation. Methylation is established by histone lysine methyltransferase and removed by histone demethylase, and it is associated with transcriptional activation or inhibition (Berger, 2007; Kouzarides, 2007; Shahbazian and Grunstein, 2007; Liu et al., 2010; He et al., 2011). Histone lysine methylation is different in terms of the number of methyl groups, such as monomethyl, dimethyl, or trimethyl lysine. The heterosis of *Arabidopsis* is related to the degree of histone modification enriched in the promoter regions of *CIRCADIAN CLOCK ASSOCIATED 1* (*CCA1*) and *LHY*. *CCA1* and *LHY* are two genes that can regulate the circadian clock process. *CCA1* and *LHY*, as well as their regulators *TOC1* and *GI*, are positively correlated with the levels of histone H3-Lys 9 acetylation (H3K9ac) and histone H3-Lys 4 dimethylation (H3K4me2). Changes in the degree of H3K9ac and H3K4me2 modification can directly lead to the decrease in *CCA* and *LHY* expression, promoting the expression of downstream genes that control photosynthesis and starch metabolism, thereby increasing the photosynthetic efficiency, starch accumulation, and growth advantage of F1 (Ni et al., 2009). *CCA1* is also a key gene in plant disease resistance heterosis. When pathogens attack, the expression of *CCA1* in F1 hybrids is accurately regulated by its rhythmic histone modification at different time points during the day (Yang et al., 2021). The expression level of *TOC1* is related to the time regulation of histone H3 acetylation. The expression peak of TOC1-luciferase report can be induced by the inhibition of histone deacetylases by trichostatin A (Perales and Mas, 2007). Li et al. (2011) established that histone modification plays a role in the pattern of changes in non-additive expression in hybrid rice by overexpression and inactivation of histone deacetylase coding genes in hybrid rice. Histone modification is allele-specific in hybrids. For example, in rice hybrid, ASE is primarily regulated by allele-specific histone modifications–histone H3 lysine 36 rather than histone H3 lysine 27 (H3K27me3) (Guo et al., 2015). Parent specificity and tissue specificity are also observed in hybrids. For example, parent-specific differences in H3K27me3 have been detected in *Arabidopsis* endosperm, especially in transposons (Moreno-Romero et al., 2016). However, the activity of panicle primordia meristem in rice is directly related to panicle development and grain yield. In *Arabidopsis* allotetraploids, most non-additively expressed genes (Wang et al., 2006), including circadian clock genes (Ni et al., 2009), are related to high levels of H3K9ac and H3K4me3 (Ha et al., 2011).

### Small RNA and Plant Heterosis

Aside from DNA methylation and histone modification, small RNA, including small interfering RNAs (siRNAs), microRNAs (miRNAs), and *trans*-acting ta-siRNAs, is also an important part of epigenetics. *HUA ENHANCER 1* (*HEN1*) is a gene-encoding RNA methyltransferase and a key factor in the biological occurrence of small RNAs in plants. The production of functional small RNA can be inhibited by the mutations of *HEN1*, and it will inhibit the growth vigor of *Arabidopsis* hybird (Shen et al., 2012). Shen et al. (2017) found that the non-additive effect of miRNA in hybrid offspring can affect the expression of target genes and regulate heterosis. And this view is supported in the research of maize and rice (Zhang et al., 2014; Zhao et al., 2015). Chen et al. (2010) conducted miRNA microarray to compare the expression levels of miRNA and the high abundance small RNAs in rice hybrid combinations. They confirmed that miRNAs are involved in regulating gene differential expression in hybrids. He et al. (2010) further found that the differential expression of miRNAs is negatively correlated with the differential expression of its target genes between the hybrid rice and its parents. While Crisp et al. (2020) suggested that hybridization affect the expression of small RNAs at specific sites, and small RNAs show more variations between inbred lines compared with gene expression variations. In addition, Hu et al. (2021) stated that 32.5% of differential alternative splicing contains or lacks at least one annotated binding site of maize miRNA in the maize hybrid and parents. They suggested that it may be involved in miRNA-mediated post-transcriptional regulation. The upregulated expression of miR156, miR159, and miR319 in hybrid offspring can regulate the target genes *SPL*, *MYB*, and *TEOSINTE BRANCHED 1/CYCLOIDEA/PROLIFERATING CELL FACTOR* (*TCP*), suggesting that they can change the plant morphology of *B. napus* to adapt to the environment. The accumulation of *MYB* mRNA can be reduced by the overexpression of miR159 and may result in male sterility, whereas the overexpression of miR159-resistant *MYB33* results in leaf curling up, dwarfing, and petiole shortening (Alonso-Peral et al., 2010). The developmental processes of leaf size, leaf shape, and flower symmetry are guided by *TCP* transcription factors (Danisman et al., 2012). Overexpression of miR319 results in inconsistent leaf shape and delayed flowering, whereas *TCP* mRNA can be specifically downregulated by miR319 (Shen et al., 2017). Overexpression of miR408a improves leaf area, petiole length, plant height, flower size and silique length in *Arabidopsis*, as well as increases photosynthetic rate in hybrid maize, resulting in high biomass and seed yield (Hou et al., 2020). miR164, miR166, miR167, and miR390 have been shown to target *ARF* genes and are involved in the IAA response pathway (Mallory et al., 2005). These miRNAs are highly conserved in plant species and their targets in *B. napus* (Shen et al., 2015). The sequencing results of a small RNA library of 21 maize inbred lines showed that variations in parental small RNA expression are negatively correlated with the heterosis of grain yield (Seifert et al., 2018). The expression of Pol IV-dependent siRNA is also negatively correlated with the expression of a group of *AGMADS-like* (*AGL*) genes, which encode type I MADS-box transcription factors and are expressed in endosperm and involved in regulating seed size (Lu et al., 2012; Chen, 2013).

In conclusion, these epigenetic modifications are of great significance to explain heterosis, especially DNA methylation, small RNA and histone modification. Generally speaking, the methylation level of hybrids with heterosis is higher than the average methylation level of parents, but not all high levels of methylation have a role in heterosis. Heterosis has a strong correlation with the change of methylation level and pattern of specific sites. The differential expression of small RNAs in hybrids and their regulatory effects on target genes may affect the biomass and grain yield of hybrids to varying degrees. Some histones found in hybrids may be related to heterosis, such as H3K9ac, H3K4me2, and H3K4me3. And different types of histone modification can regulate genes related to photosynthesis, circadian rhythm and metabolic level, leading to the heterosis in hybrids. However, due to the large number of histones and the variety of their modification methods, the regulatory mechanism between histones and heterosis needs further research.

## Hormone Regulation and Plant Heterosis

DEG-targeted hormone-mediated signaling pathways mainly involve abscisic acid, jasmonic acid, salicylic acid, brassinosteroids, and auxin. These hormones have been shown to play an important role in heterosis formation (Li Y. et al., 2016; Zhang et al., 2016; Hu et al., 2017). Pathways involved in various biological and abiotic stresses have also been confirmed to participate in heterosis (Groszmann et al., 2015; Miller et al., 2015; Wang et al., 2015). Ethylene is a plant hormone that promotes fruit maturation but inhibits hypocotyl elongation. The application of exogenous ethylene can eliminate the biomass activity of *Arabidopsis* F1 hybrids (Song et al., 2018), and it plays a negative role in heterosis and salicylic acid can inhibit *ETHYLENE RESPONSE FACTORS* (Shen et al., 2017). The monosaccharide transporter gene is downregulated by the overexpression of the ethylene receptor gene *ETR2* in rice, thereby preventing the sugar from transferring from stem to grains, resulting in the reduction of grain weight (Wuriyanghan et al., 2009). Consistent with these studies, Katara et al. (2020) also observed the downregulation of the ethylene receptor gene *ETR* in two hybrid sterile lines. In WFYT025 hybrid rice, regulatory changes in gibberellin and abscisic acid biosynthetic genes can lead to heterosis, and it might have a stronger gibberellin biosynthesis potential than its parents, thereby promoting the increase in spikelet primordium number of the hybrid WFYT025. Moreover, the phosphorylation of SNF1-related protein kinase 2 is necessary for the kinase activity of downstream targets, which is related to abscisic acid biosynthesis (Chen L. et al., 2018). Smith (2019) noted that a decrease in the salicylic acid level in hybrids with abnormally high salicylic acid level would promote the hybrid’s growth. By contrast, the salicylic acid-regulated defense genes of hybrids that show heterosis are downregulated. Both *Arabidopsis* C24/Ler F1 hybrids and C24 bacterial degradative enzyme salicylate 1 hydroxylase may promote changes in the main regulatory factor *TL1 BINDING TRANSCRIPTION FACTOR 1* (*TBF1*) for defense and growth by regulating the level of salicylic acid, thereby promoting plant growth (Gonzalez-Bayon et al., 2019). Hydrogen peroxide (H_2_O_2_) is also an important signal molecule that can interact with different plant hormone signaling pathways, such as abscisic acid, salicylic acid, jasmonic acid, auxin, and brassinosteroids; regulate plant development and stress response (Saxena et al., 2016; Yuan et al., 2017); and induce the oxidation of *BRASSINAZOLE-RESISTANT 1* (*BZR1*) transcription factor, a major regulator of the brassinosteroid signaling pathway. H_2_O_2_ oxidation of *BZR1* enhances its interaction with *PHYTOCHROME-INTERACTING FACTOR 4* (*PIF4*) and *AUXIN RESPONSE FACTOR 6* (*ARF6*) (Tian Y. et al., 2018). The upregulation of the auxin biosynthesis gene *YUCCA* (*YUC*) can increase the level of IAA, which in turn can be activated by the transcription factor *PIF4* (Sun et al., 2012, 2013; Wang et al., 2017). The yield heterosis for improving the plant structure of *B. napus* can be enhanced by the degradation mutation of the potential gene *BnaA3.IAA7* that encodes auxin/indole acetic acid proteins (Li H. et al., 2019). *TCP4* directly activate *YUC5* transcription, all of which integrate organ morphogenesis with auxin and brassinosteroid reactions that promote hypocotyl cell elongation. In addition, *TCP4* requires brassinosteroid response to promote hypocotyl growth (Reddy et al., 2016). These studies confirmed the contribution of epigenetic regulation of hormone signals and gene expression to heterosis. However, hormone level is only a part of plant growth and heterosis.

As we all know, plant growth is inseparable from hormones. In recent years, great progress has been made in the study of plant hormones involved in plant growth and development, including gibberellin promoting plant height, abscisic acid promoting seed germination, and the interaction between hormones leading to the changes of reproductive organ number and grain weight. At the same time, these traits show relatively obvious heterosis, but hormone regulation is effective in a certain range. If the hormone level is too high, it will have a negative effect. Therefore, the differences in metabolism and regulation of plant hormones between parents and hybrid progenies may become a research hotspot in the future, which will be conducive to further analysis of the mechanism of plant heterosis.

## Regulation Network of Biomass Heterosis and Grain Heterosis

Yield is the top priority of plant production. Breeders pursue different yields for different plants. In some plants, they focus more on biomass yields, such as leafy vegetables and graminaceous crops. Some pursue root and tuber yield, such as potato and sweet potato. However, people may pursue different yields within the same crop, such as grain yield for conventional maize and biomass yield for forage silage maize.

Cell division and photosynthesis of hybrid plants are important components of growth vigor (Liu et al., 2021). Leaf growth in early development is the key to biomass heterosis in *Arabidopsis*. However, heterosis is not caused by the increase in photosynthetic efficiency per leaf area but by the increase in total leaf area and total photosynthesis per plant (Liu P. et al., 2020). In addition to photosynthesis and sucrose and starch pathways, oxidative phosphorylation and the tricarboxylic acid cycle may also play a role in heterosis (Yao et al., 2005). The yield and biomass heterosis of hybrids may be due to altered expression patterns of genes that control biological functions (such as carbon fixation, sugar metabolism and circadian rhythm) (Chen, 2013). Dry matter yield, which represents the biomass of barley, is closely related to grain yield. Grain yield is mainly determined by additive genetic effect (Zhang et al., 2015), while dry matter yield is affected by both additive and non-additive genetic components (Madić et al., 2014). Singh et al. (2013) found that dry matter yield could also be affected by general combining ability and special combining ability. And Mühleisen et al. (2013) found that special combining ability has a significant effect on grain yield. Similarly, in cultivated tomato, biomass and yield are correlated in terms of heterosis (Semel et al., 2006). In addition, ploidy effect is also related to biomass and grain yield heterosis. Studies have shown that the effect of ploidy on plant size seems to have a dosage compensation mechanism (Miller et al., 2012). In maize, plant size increases with increasing ploidy (from haploid to triploid), but decreased in tetraploid (Riddle et al., 2006). In the study of *Arabidopsis*, the effect of ploidy on biomass is not obvious, but the increase of ploidy level is positively correlated with seed size and weight (Miller et al., 2012).

Rice, which represents graminaceous plants, show substantial heterosis in economic yield, especially in grain yield. In plant breeding, the most effective study of heterosis has been conducted in rice. Rice yield and quality have been greatly improved. In 2017, the yield of the super hybrid rice reached the peak of 1149 kg/666.67 m^2^ (Li et al., 2020). The grain yield heterosis of rice is controlled by *Hd3a*; *TAC1* of CMS lines; *LAX1* and *Ghd8* of the environment-sensitive Indica hybrid; and *Sd-1*, *GW6a*, *DEP1*, *IPA1*, *NAL1*, and *N11q25* of the Indica × Japonica rice. *Ghd8* is the main heterosis gene in most Indica–Indica hybrid rice systems and also the primary heterosis gene in the yield of LYP9 (Huang et al., 2016; Li D. et al., 2016). Early flowering of hybrid rice can be caused by the key genes *Ghd7* and *Hd3a* (Li et al., 2011), whereas *Ghd7* encode a CCT (CO, CO-like, and TOC1) domain protein, and they play a key QTL role in controlling rice yield, plant height, and heading date, *Ghd7* is also expressed under long-day conditions, inhibiting the expression of *Early heading date 1* (*Ehd1*), thereby inhibiting long-day flowering (Xue et al., 2008). Overall, rice grain yield is most affected by the flowering time genes *SOC1*, *Ghd8*, and *Ghd7* (Tadege et al., 2003; Xue et al., 2008; Yan et al., 2011). *Heading date 1* (*Hd1*), *CONSTANS* (*CO*) homolog of *Arabidopsis* in rice, activates *Hd3a* [a homolog of *FLOWERING LOCUS T* (*FT*)] under short-day conditions and inhibits *Hd3a* under long-day conditions (Yano et al., 2000; Hayama and Coupland, 2004). *Ehd1* can up-regulate *Hd3a* expression and promote short-day-dependent flowering in rice, which is inhibited by *GI*. Furthermore, *Ehd1* can activate *RICE FLOWERING LOCUS T1*. In rice, overexpression of the histone deacetylase gene *HDT1* can repress the non-additive expression of *Hd1* and *GI* in the hybrid (Komiya et al., 2009; Li et al., 2011; Doi et al., 2016). A highly conserved R2R3 *MYB* domain transcription factor family is encoded by the gibberellin- and abscisic acid-regulated *MYB* (*GAMYB*) or *GAMYB-like* genes, in which the expression of *GAMYB* is induced by gibberellic acid (Woodger et al., 2003; Aya et al., 2009; Murray et al., 2010). Moreover, *GAMYB* is negatively regulated by the miRNA family *miR159* (Tsuji et al., 2006; Alonso-Peral et al., 2010).

Heterosis in biomass yield is represented by the model plant *Arabidopsis*. Previous studies on *Arabidopsis* heterosis revealed potential heterosis phenotype candidate genes in circadian clock, flavonoid biosynthesis, auxin transport, salicylic acid metabolism, and response pathways (Ni et al., 2009; Shen et al., 2012; Groszmann et al., 2015; Zhang et al., 2016; Lauss et al., 2018). Early seedling biomass heterosis is related to the earlier expression in photosynthetic pathway relative to the parents, and high IAA levels may be attributed to the early photosynthesis of hybrid seedlings (Zhu et al., 2020). The transcription factor *PIF4* also plays an important role in heterosis (Wang et al., 2017). *DDM1* affects *Arabidopsis* heterosis in seedlings by regulating salicylic acid metabolism (Zhang et al., 2016). The metabolic level of salicylic acid can promote changes in the main regulatory factor *TBF1* of defense and growth, thereby promoting plant growth (Gonzalez-Bayon et al., 2019). The changes in the expression of time gene *CCA1* can enhance carbon and starch accumulation and promote the formation of heterosis in biomass (Ko et al., 2016). Epigenetic activation of *CCA1* can also promote salicylic acid accumulation in hybrids; thus, heterosis works in defense (Yang et al., 2021). *CCA1* and *LHY* regulate the biomass clock process, and their regulators *TOC1* and *GI* are positively correlated with the levels of H3K9ac and H3K4me2 (Ni et al., 2009). In the morning-phased loop, *LHY* and *CCA1* activate the expression of *Pseudo-response Regulator 7* (*PRR7*) and *PRR9* genes, which maintain period length and amplitude in plants (Shahzad et al., 2020). Downregulation of *CCA1* during the daytime indirectly inhibits the expression of *1-aminocyclopropane-1-carboxylic acid synthase* (*ACS*) in hybrids to reduce ethylene production. *PIF4* and *PIF5* usually activate the expression of *ACS* at night (Song et al., 2018). The transcription factors *LEAFY* (*LFY*) is upregulated by *FT* through *SOC1* in *Arabidopsis* (Das et al., 2019). *LFY* and *FUL* are directly activated by the miRNA-targeted transcription factor *SQUAMOSA PROMOTER BINDING PROTEIN-LIKE 3* (Yamaguchi et al., 2009). *GAMYB* transcription induces the expression of *LFY* (Achard et al., 2004). In addition, in the study of miRNA, MiR160 is complementary to *AUXIN RESPONSE FACTOR 10* (*ARF10*), and miR167 is complementary to *ARF6* and *ARF8* (Rhoades et al., 2002).

**FIGURE 1:**
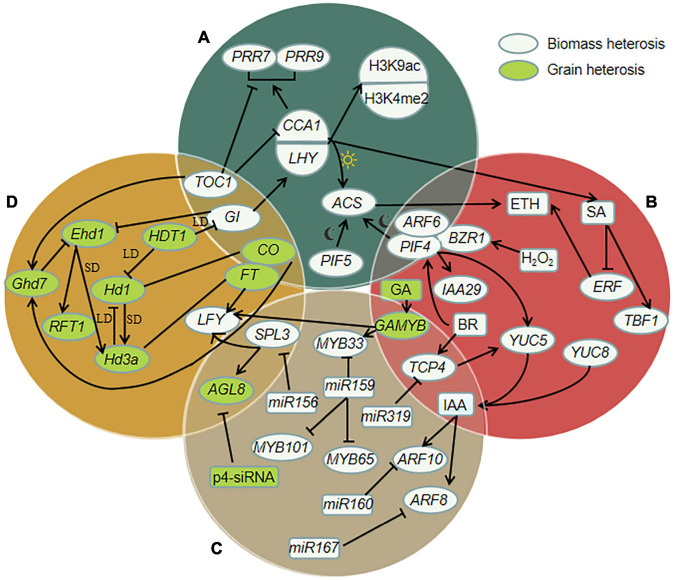
Schematic diagram of molecular regulation in biomass and grain heterosis. Solid arrows represent positive regulation, inhibitory arrows represent negative regulation; straight lines represent homology. **(A)** Heterosis regulation by photoperiod rhythm related genes. *CIRCADIAN CLOCK ASSOCIATED 1* (*CCA1*) and *LATE ELONGATEDD HYPOCOTYL* (*LHY*) and their regulators *TIMING OF CAB EXPRESSION 1* (*TOC1*) and *GIGANTEA* (*GI*) are positively correlated with histone H3-Lys 9 acetylation (H3K9ac) and histone H3-Lys 4 dimethylation (H3K4me2). *LHY* and *CCA1* can activate the expression of *PSEUDO-RESPONSE REGULATOR 7* (*PRR7*) and *PRR9*, and *TOC1* can inhibit the expression of *PRR7* and *PRR9*. *PHYTOCHROME-INTERACTING FACTOR 4* (*PIF4*) and *PIF5* can often activate expression of *1-aminocyclopropane-1-carboxylic acid synthase* (*ACS*) (ai night), down-regulation of *CCA1* inhibites the expression of *ACS* (at daytime), and *ACS* can promote the increase of ethylene. **(B)** Regulation of heterosis involving hormones and related genes. *CCA1* can promote the increase of salicylic acid (SA) in hybrids. SA promotes the expression of *TL1 BINDING TRANSCRIPTION FACTOR 1* (*TBF1*) and inhibites the expression of *ETHYLENE RESPONSE FACTORS (ERF)*. Hydrogen peroxide (H_2_O_2_) and brassinosteroid (BR) enhance the interaction of *BRASSINAZOLE-RESISTANT 1* (*BZR1*) with *PIF4* and *AUXIN RESPONSE FACTOR 6* (*ARF6*). *PIF4* can activate the expression of *YUCCA8* (*YUC8*) and *IAA29*, and the up-regulation of *YUC* genes leads to the increase of IAA level. *TEOSINTE BRANCHED 1*, *CYCLODEA*, *PROLIFERATING CELL FACTORS 4* (*TCP4*) directly activates the expression of *YUC5*, and the reaction of BR is helpful for the expression of *TCP4*. The expression of *gibberellin- and abscisic acid-regulated MYB* (*GAMYB*) is induced by GA and negatively regulated by miR159, and *GAMYB* can promote the expression of *LEAFY* (*LFY*) and *MYB33*. **(C)** Regulation of heterosis involving small RNA and related genes. Overexpression of microRNA159 (miR159) can inhibit *MYB33*, *MYB65*, *MYB101*. MiR319 can inhibit *TCP4*. MiR160 targets *ARF10*, miR167 targets *ARF8* genes, and they are regulated by IAA. The expression of Pol IV-dependent siRNAs (p4-siRNA) is correlated with the expression of *FRUITFULL* (*AGL8, also known as FUL*). And the expression of *AGL8* is inhibited by *SQUAMOSA PROMOTER BINDING PROTEIN-LIKE 3* (*SPL3*), *SPL3* can be inhibited by miR156. **(D)** Heterosis regulation by flowering related genes. *Grain number, plant height and heading date 7* (*Ghd7*) can be promoted by *CONSTANS* (*CO*) and *TOC1*. *Heading date 1* (*Hd1*, *CO* homologous genes) and *Early heading date 1* (*Ehd1*) up-regulate the expression of *Heading date 3a* (*Hd3a*, *FLOWERING LOCUS T*, *FT* homologous genes) under short-day (SD) conditions. Under long-day (LD) conditions, the expression of *Ehd1* is inhibited by *Ghd7*, while *Hd1* is the inhibitor of *Hd3a*, histone deacetylase gene *HDT1* inhibites *Hd1* and *GI*. Inhibition of *GI* can promote the expression of *Ehd1*. And the increase of *Ehd1* induces the expression of *RICE FLOWERING LOCUS T1* (*RFT1*). *SPL3* and *FT* can promote the expression of *LFY*.

Heterosis is a complex biomass phenomenon. Numerous studies have shown that the strength and formation mechanism of heterosis of different biological species, different varieties of the same crop, and different traits may be different. The key to heterosis formation lies in the genetic differences between parents. The genomic sequence information of hybrids from parents does not change, and the phenotype and protein and gene expression substantially change. The orderly expression of many genes under the action of various regulatory factors forms a network system that affects the heterosis of grain yield and biomass yield by regulating different ways, as shown in Figure 1.

## Concluding Remarks and Future Perspectives

In recent years, changes in gene expression levels and expression patterns between hybrids and parents have been compared *via* molecular biology and molecular genetics techniques. The DEGs obtained by these analyses are mainly focused on photosynthesis, carbohydrate metabolism, and energy metabolism. However, the molecular mechanism of heterosis formation cannot be explained well by these processes. Heterosis formation is a complex process that is affected by various factors, such as genes, environment, and the expression regulation of several genes related to physiological metabolism. Gene expression is characterized by spatiotemporal expression, but it is also affected by the surrounding environment. The material basis of heterosis is the hybridization of genotypes and not the simple embodiment of the overall heterozygosity between two parents. In terms of epigenetics, the issue of whether circular RNAs and long non-coding RNAs participate in heterosis remains unresolved. With the development of biological technology, an increasing number of multi-omics methods have been adopted to analyze problems in heterosis. Heterosis is a complex trait that is regulated by multiple genes. Therefore, genomics, transcriptomics, proteomics, metabolomics, phenomics, and ionomics can be possibly combined to analyze effective genes. Comprehensive analysis from multiple levels helps to better understand metabolic networks, gene functions, biochemical pathways and their correlations, and establish the interdependence between different cell components to better describe molecular phenomena. These results will provide support for revealing the molecular genetic mechanism of plant heterosis. In addition, the use of multi-omics combined with high-throughput tools can bring revolutionary changes to plant biology, because it provides real-time readings of hundreds of genes, proteins, metabolites and ions at different developmental stages and different environmental conditions, which is bound to promote the breakthrough in molecular theory of heterosis breeding. The agronomic traits of hybrids are better than those of parents. Hence, the selection of parental combinations is also a challenge, which may have general heterosis, MPH, and BPH. In the study of heterosis, finding the main QTLs or genes that regulate metabolism will be a hot topic. In addition, the rise of research on bacterial defense heterosis (Yang et al., 2021), stock heterosis (Asaf et al., 2021), sterile line gene editing systems, MiMe (Cas9) systems, and even new biotechnology approaches (Yu et al., 2021) has aroused the interest of researchers involved in heterosis research.

## Author Contributions

XW wrote the manuscript and YL assisted the work. RG and YZ designed the review. RG was responsible for the revision of this manuscript. All authors contributed to the article and approved the submitted version.

## Conflict of Interest

The authors declare that the research was conducted in the absence of any commercial or financial relationships that could be construed as a potential conflict of interest.

## Publisher’s Note

All claims expressed in this article are solely those of the authors and do not necessarily represent those of their affiliated organizations, or those of the publisher, the editors and the reviewers. Any product that may be evaluated in this article, or claim that may be made by its manufacturer, is not guaranteed or endorsed by the publisher.
